# Apophyseal ring fracture associated with two levels extruded disc herniation: case report and review of the literature

**DOI:** 10.1590/S1679-45082014RC2736

**Published:** 2014

**Authors:** José Alexandre Lopes da Silva Alvarenga, Fernando Tadashi Salvioni Ueta, David Del Curto, Renato Hiroshi Salvioni Ueta, Delio Eulalio Martins, Marcelo Wajchenberg, Eduardo Barros Puertas

**Affiliations:** 1 Escola Paulista de Medicina, Universidade Federal de São Paulo, São Paulo, SP, Brazil

**Keywords:** Spinal fractures/diagnosis, Spinal fractures/surgery, Intervertebral disc displacement/diagnosis, Intervertebral disc displacement/surgery, Case reports

## Abstract

Apophyseal ring fractures are rare injuries that may be associated with lumbar disc herniation in young patients. We report a unique case in the literature of a 15-year-old male patient who played football and was admitted at our service complaining of sciatica radiating into the left leg. An apophysial ring injury of L5 vertebral body was observed. This injury caused two extruded disc herniation in adjacent levels. Surgical procedure was indicated after failure of conservative treatment.

## RESUMO

As fraturas do anel apofisário são lesões raras, que podem estar associadas à hérnia de disco lombar nos pacientes jovens. Apresentamos aqui um caso único na literatura de paciente de 15 anos, gênero masculino, praticante de futebol, com queixa de ciatalgia para o membro inferior esquerdo. Houve lesão do anel apofisário, nos planaltos superior e inferior do corpo vertebral de L5, ocasionando hérnias discais extrusas nos níveis adjacentes. O procedimento cirúrgico foi indicado após a falha do tratamento conservador.

## INTRODUCTION

Apophyseal ring fractures are rare injuries that can occur without noticeable symptoms.^(1)^ In 1973, Lowrey was the first to suggest the theory of posterior apophyseal avulsion.^(2)^ These injuries are caused by microtraumas due to repetitious activities and often affect adolescents and young adults who apophyseal ring and vertebral body are incompletely fused before 18 years old.^(3)^ There are reports stating that most affected sites are vertebral bodies L4 and L5.^(4–6)^ Although the association between apophyseal ring and herniated disc is described in young patients, its mechanism is uncertain.^(7–9)^ It is well know that in these cases, disc herniation can occur without annulus fibrosus injury.^(10)^

This paper reports a unique case in the literature of injury in apophyseal ring in upper vertebral plateau L5 that caused extruded disc herniation in adjacent levels (L4-L5 and L5-S1).

## CASE REPORT

A 15-year-old male patient who trained football five days a week reported suffer of lower back pain and sciatica radiating into his lower left limb for 1 year. The condition affected his ability to exercise, and no traumatic events during this period were reported. Because pain intensity increased in last 6 months, the patient was referred to our service for assessment.

At the initial physical examination he had spasms of paravertebral musculature, ischiotibial muscle shortening, positive result for left side in straight-leg-raising test of 40° and contralateral pain when test was conducted in the right side that presented 50° of leg raising and absence of neurologic deficit.

**Figure 1:**
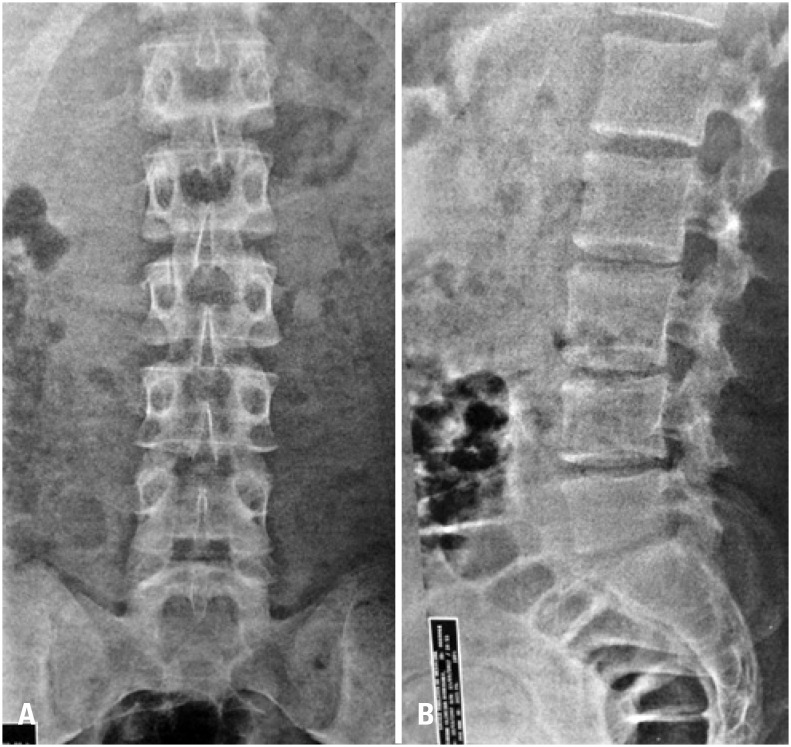
(A) Posteroanterior radiographs of the lumbar sacral spine. (B) Lateral radiograph of lumbar sacral spine

Radiographic exam showed a mild antalgic scoliosis in the anterior-posterior incidence in lumbosacral spine (Figure 1A). In lateral incidence, we observed short pedicles and apparent shortening of height in posterior region of vertebral body L5 (Figure 1B).

**Figure 2:**
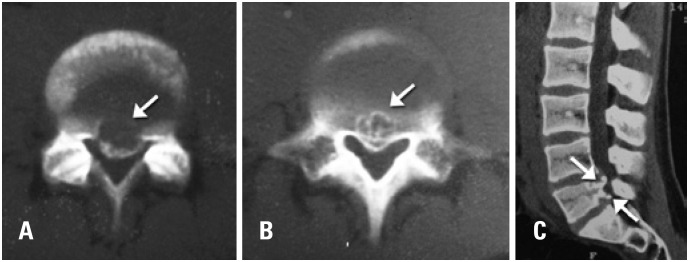
(A and B) Computed tomography and axial slices showing the apophyseal ring fracture (arrow). C) Sagittal slice shows the double injury of apophyseal ring (arrows)

A computed tomography was requested to confirm the diagnosis (Figure 2) and the result showed an avulsed bone fragment from the posterior plateau of vertebral body L5, posteriorly displaced and with signs of sclerosis. In CT it was also possible to observe two central-lateral disc herniations, extruded and migrated in levels L4-L5 and L5-S1, which were detailed in magnetic resonance.

**Figure 3:**
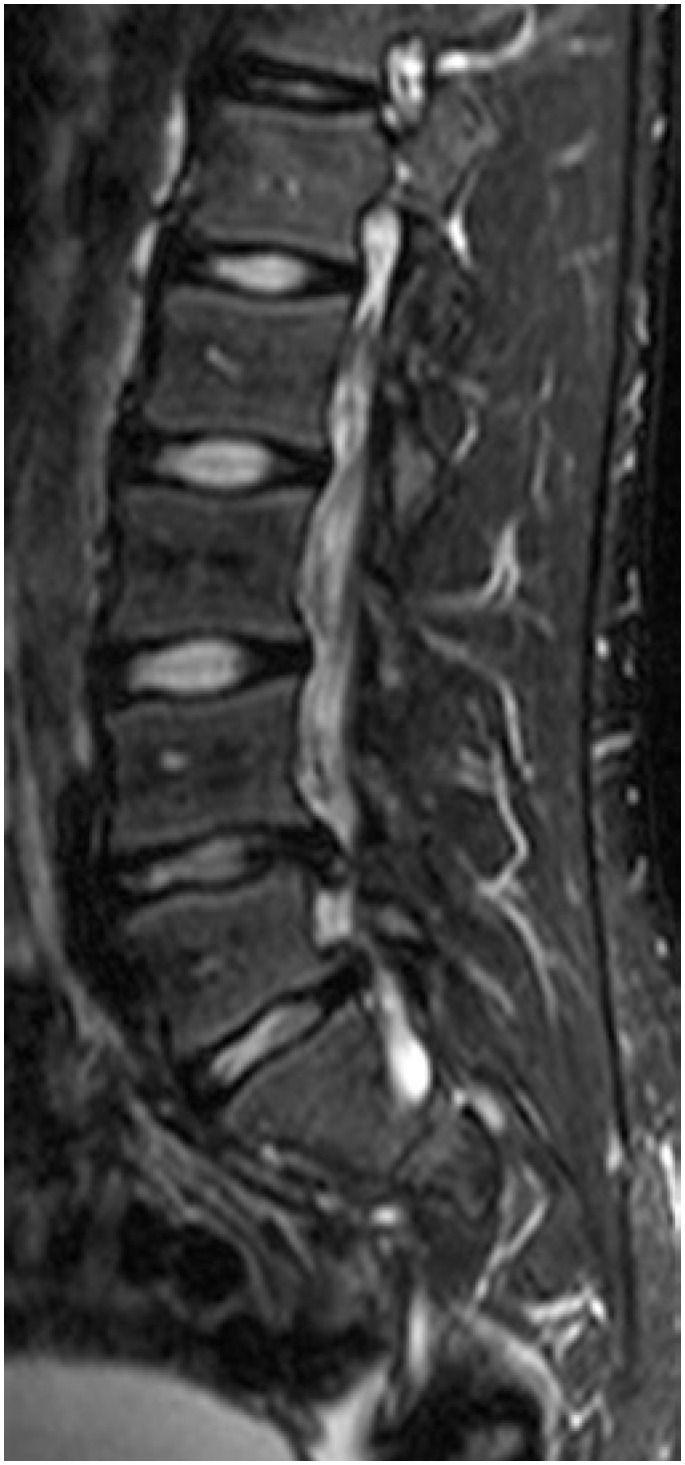
Magnetic resonance, sagittal slice, weighted in T2, showing two extruded herniated disks in levels L4-L5 and L5-S1

In magnetic resonance of lumbosacral spine in sagittal cut and weighted in T2, two extruded disc hernia in levels L4-L5 and L5-S1 were seen (Figure 3). In L4-L5 level the hernia was migrated to caudal whereas the space L5-S1 was migrated to cephalic region.

The patient underwent non-surgical treatment for 6 weeks with analgesic, non-steroids anti-inflammatories, physiotherapy and resting, but clinical picture did not improve. Because he evolved with paresthesia in L5 and S1 dermatomes and loss of muscular strength in lower left limb in the same myotomes, surgical treatment was indicated.

**Figure 4:**
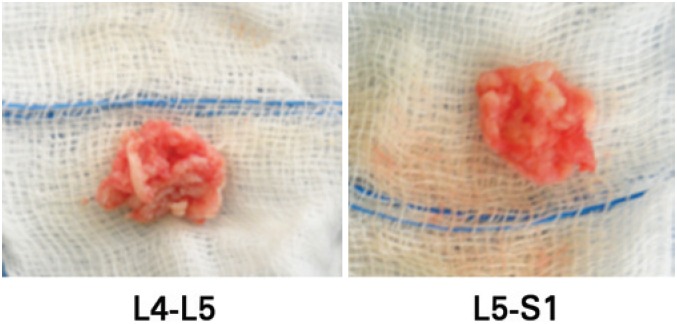
Disc material removed in the surgical procedure

The resection of herniated discs was conducted by two laminectomies in left side in the lower portion of L4-L5 lamina removed from the yellow ligament of the lateral recess opening. This procedure enables to view the descending branches that were hypermediated and compressed by bulging discs, however, avulsed bone fragments were not seen during the surgery. The disc material was removed and then it was presented (Figure 4).

The patient was discharged within 24 hour after the surgery. After, he started complementary treatment with physiotherapy for 7 days. During the first month after the surgery, the patient reported gain of strength in the lower left limb, absence of parenthesis and negative result in straight-leg-raising test.

## DISCUSSION

Apophyseal ring fractures is a rare injury^(11)^ that affects the posterior region of vertebral body L4 and L5 in more than 90% of cases. It is more prevalent in male adolescents and young adults.^(1,12)^ However, a recent retrospective study showed that this injury is also found in adults and, in those patients, the most affected level was the L5-S1, especially the upper plate of S1.^(13)^ In all cases reviewed in the literature, the apophyseal injury caused a single disc herniation. Our case is the unique reported in the literature because the apophyseal ring injury caused two extruded disc herniation in the adjacent levels. The most common symptoms are low back pain and radicular pain, but neurologic deficit is rare.^(14)^ Other symptoms include limp, paravertebral muscle spasm and shortening of ischiotibial muscle.

Sports-related microtraumas are considered the main etiology,^(15)^ because they lead to progressive avulsion of apophyseal ring and cause the deviation of the fragment in direction to vertebral canal. There is no consensus concerning the injury associated with intervertebral disc.^(10,11)^

This disease physiopathology is explained by ossification of apophyseal ring between 4-6 years of age and its fusion at roughly 18 years of age. This is firmly adhered to fibrous annulus by Sharpey's fibers and to some fibers of posterior longitudinal ligament. Therefore, microtraumas due to repetitious activities can lead to extraction of the apophyseal ring that is incompletely fused so that causing the injury.

The diagnosis of the apophyseal ring fracture requires a detailed physical exam, associated with complementary exams. The simple radiography gives few information and presents isolated accuracy that ranges from 29% to 69%.^(12,16,17)^ The computed tomography is the ideal exam to visualize the avulsed bone fragment. However, the magnetic resonance also enables fragment evaluation, besides showing the quality of intervertebral disc and herniated disc, without the need to expose the patient to ionizing radiation.

In this report, it was important to exclude other possible diagnoses such as spondylolysis, infectious process, tumors and fractures.

Takata et al.^(17)^ proposed a classification that is subdivided in three types based in tomographic findings. The type I corresponds to simple separation of posterior vertebral margin without bone defect; type II is the fracture by posterior margin avulsion of vertebral body; and type III consists in the small posterior fracture to a cartilaginous irregularity of motor plate. Epstein and Epstein^(18)^ described the type IV with a complete dislocation of the vertebral body posterior wall.

The initial treatment consists in analgesia, change of activities or interruption of physical activity, non-steroids anti-inflammatory and lumbar orthosis. When the fragment is reabsorbed or whether it will cause an extensive ossification is unknown. The indication of surgical decompression consists the gap in the conservative treatment with persistent lumbar pain that affects the functional ability of patient, with or without neurologic deficit. In rare cases with neurologic deficit the surgical treatment is indicated with no delay.^(14)^

The surgical proposed involves laminectomy and discectomy, but excision of bone fragment is controversial.^(14,19)^ In several situations, the fragment is not seen and the injury could appear as a simple disk protrusion.^(19)^ If the bone fragment does not cause compression, there is no need for excision because it will increase the time and surgical risk because the mentioned fragment is located ventrally to dural sac, and its approach will cause more harm than good. In addition, the resection of the fragment does not influence the clinical results and it is not always necessary to achieve satisfactory outcomes.^(9)^ Epstein emphasized that when the fragment is fused, only the decompression can be conducted.^(20)^

A recent literature review^(21)^ highlighted that surgeons should consider the need of decompression, removal of the fragment and fusion of the segment involved. In addition, it suggests that each case should be evaluated in an independently manner.

## CONCLUSION

The surgical results are often favorable and agree with the case in our report, which presented improvement in motor strength and gradual return to physical activity. Because of the short follow-up period and problems in methods of selected studies, it was not possible to conclude what is the best modality of treatment.
